# Cohort Profile: The Stroke in Sierra Leone (SISLE) Register

**DOI:** 10.1093/ije/dyad112

**Published:** 2023-08-09

**Authors:** Daniel Youkee, Iain J Marshall, Julia Fox-Rushby, Durodami R Lisk, Jessica O’Hara, Yanzhong Wang, Anthony Rudd, Charles D A Wolfe, Gibrilla F Deen, Catherine Sackley

**Affiliations:** King’s School of Life Course and Population Sciences, King’s College London, London, UK; King’s School of Life Course and Population Sciences, King’s College London, London, UK; King’s School of Life Course and Population Sciences, King’s College London, London, UK; College of Medicine and Allied Health Sciences, University of Sierra Leone, Freetown, Sierra Leone; King’s School of Life Course and Population Sciences, King’s College London, London, UK; King’s School of Life Course and Population Sciences, King’s College London, London, UK; King’s School of Life Course and Population Sciences, King’s College London, London, UK; King’s School of Life Course and Population Sciences, King’s College London, London, UK; College of Medicine and Allied Health Sciences, University of Sierra Leone, Freetown, Sierra Leone; King’s School of Life Course and Population Sciences, King’s College London, London, UK

Key Features

- The Stroke in Sierra Leone (SISLE) register recruited all patients aged 18 years and over and presenting with the World Health Organization definition of stroke at the two principal adult tertiary hospitals in Freetown, Sierra Leone: Connaught Teaching Hospital and 34th Military Hospital. Patient charges for hospital admission and investigations, including neuroimaging, were paid by the study to reduce the cost barrier to accessing care and to minimize selection bias onto the register.
- All patients underwent standardized sociodemographic and risk factor assessment, followed by the measurement of National Institute of Health Stroke Scale (NIHSS), Barthel Index, Modified Rankin Scale (mRS), and EQ-5D-3L by a trained research team, upon admission. Biochemical data collection and neuroimaging were conducted during admission. Classification of pathological stroke subtype was conducted by an experienced stroke physician, with reference to the case history, investigation results and imaging.
- From 1 June 2019 until 30 September 2021, the register recruited 986 people with confirmed stroke. Of these, 857 (87%) underwent neuroimaging. 625 (63%) were ischaemic, 206 (21%) experienced primary intracerebral haemorrhage, 25 (3%) experienced subarachnoid haemorrhage and 130 (13%) were of undetermined stroke type.
- Participants were followed up at 90 days and 1 year post stroke. Follow-up rates were 88.3% at 90 days and 81.5% at 1 year for participants alive at last point of contact.
- The SISLE anonymized dataset is available upon request to researchers, see data access section.

## Why was the cohort set up?

Stroke is the second leading cause of death in Sub-Saharan Africa (SSA),^1^ with evidence suggesting that incidence is increasing over time.^2^ However, epidemiological studies of stroke in SSA are limited in number and quality,^3^ and there are large gaps in our understanding of the natural history of stroke in SSA.^4^ Stroke is preventable, and 90% of stroke burden globally is attributable to modifiable risk factors.^5^ However, these risk factors differ by ethnicity, geographical location, socioeconomic status and access to health care.^6^^,^^7^ The Stroke in Sierra Leone (SISLE) register is a prospective observational stroke register established to determine the prevalence of stroke risk factors, stroke phenotypes, patient pathways to care, quality of hospital care and outcomes after stroke in Sierra Leone.

Stroke registers provide the data to estimate burden of disease, drive quality improvement^8^^,^^9^ and assess stroke systems of care.^10^ The register methodology is derived from the South London Stroke Register^11^ and World Health Organization STEPS methodology.^12^ The register uses standardized international criteria and scales, to provide information on stroke demographics, prevalence of risk factors, stroke care pathways, stroke types and outcomes after stroke. It is based at the two principal adult tertiary hospitals in Freetown: Connaught Teaching Hospital and 34th Military Hospital. To reduce selection bias onto the register and create a more representative cohort, all patient investigations, including neuroimaging, were provided for free to patients.

Freetown is the capital city of Sierra Leone, a low-income country in West Africa. Freetown is rapidly urbanizing, with estimated population growth of 4.2% per year.^13^ Adult life expectancy at birth is 60 years, maternal mortality rate is 717 per 100 000 live births in 2019 and under-5 mortality was 122/1000 (live births).^14^ The Sierra Leone health system is geared towards maternal and child health, with low availability of non-communicable disease (NCD) services, and low availability of drugs at health facility level for cardiovascular diseases (range 4–29%), diabetes (range 3–5%) and other NCD (risk) conditions (range 1–38%) at government facilities.^15^ Adult care for NCDs and NCD risk factors is largely funded by out-of-pocket payments by the patients and is a significant barrier to access to care and to quality care.^16^

Data from the SISLE register informed the development of the Sierra Leone Non-Communicable Diseases Strategic Action Plan 2020–2024.^17^ The SISLE register catalyzed the setup of a specialized stroke unit at Connaught Teaching Hospital in Freetown, and will be used to analyse the effect of implementing stroke unit-based care in this setting.

## Who is in the cohort?

All patients aged 18 years and over, presenting with suspected stroke, were recruited from 1 May 2019 until 30 September 2021. Stroke was defined according to the World Health Organization definition: ‘rapidly developing clinical signs of focal (or global) neurological deficit lasting more than 24 hours or leading to death, with no apparent cause other than that of vascular origin’.^18^ All stroke subtypes were included: ischaemic (ICD63), intracerebral haemorrhage (ICD61), subarachnoid haemorrhages (ICD60) and unspecified stroke types(ICD62).^19^ Multiple overlapping sources of notification were used inside the hospital including: accident and emergency admission logs; radiology request notes, ward admission logs; and physiotherapy referral logs. Daily ward rounds were performed by the clinical research team in the acute medical admission unit, and daily ward rounds of the four medical wards, private wards and the intensive care unit.

All physicians and medical nurses at the hospital were regularly briefed on the study criteria to maximize case ascertainment. To promote referral of patients onto the register, awareness of the study was raised among medical practitioners in Freetown through advertisement of the study at continuing medical education sessions, study visits to large private hospitals and regular announcement of inclusion criteria on health-related Whatsapp™ groups. Hot and cold pursuit through daily ward rounds by the study team at the hospitals to identity stroke patients, and weekly review of admission logs, were conducted to maximize case capture.^20^

In all 1145 patients were referred to the study. After review by a stroke physician and neuroimaging, 986 patients were confirmed as suffering strokes and were eligible for the study. Diagnosis of stroke and stroke subtype was made by a stroke physician (AR), after review of clinical findings, with reference to investigations including neuroimaging. A total of 857 (87%) patients underwent neuroimaging: 625 (63%) were ischaemic, 206 (21%) experienced primary intracerebral haemorrhage, 25 (3%) experienced subarachnoid haemorrhage and 130 (13%) were of undetermined stroke type. Of the cohort, 851 (86.3%) patients had first in a lifetime stroke.

## How often have they been followed up?

After the initial hospital admission, all participants were followed up at 90 days and 1 year, with subsequent annual follow-up. Participants are initially contacted by telephone and those who do not respond are visited at their homes. Follow-up was ceased due to lack of funding on 3 October 2022, follow up rates^21^ are 88.3% at 90 days, and 81.5% at one year, Table 1. Characteristics of those followed up vs those lost to follow-up at 1 year are shown in the Supplementary Table S1 (available as Supplementary data at *IJE* online). The SISLE register team are actively searching for further funding. Further funding will allow continuation of the Stroke Register, to study the impact of implementing stroke unit care, increased follow-up to study longer-term outcomes after stroke and potential extension of the register to rural health and demographic surveillance sites.

**Table 1.** Follow-up counts as of 3 October 2022

Time point	Contacted and alive	Verified alive at later time point	Total alive	Total dead	Lost to follow-up
At discharge	631		631	355	0
At 90 days	433	18	451	438	115 (11.7%)
At 1 year	308	4	312	492	182 (18.5%)

## Follow-up counts

**Figure 1.**
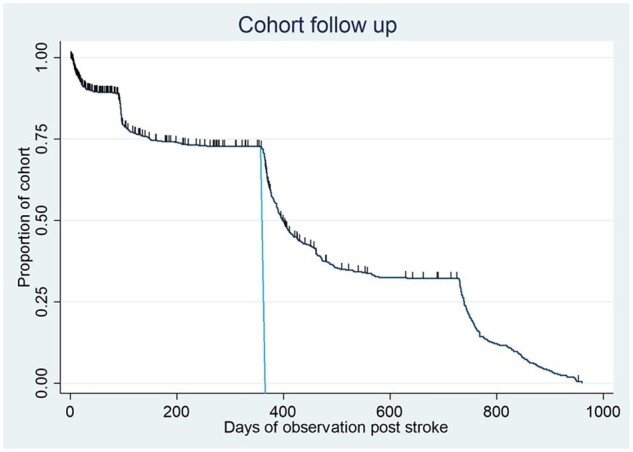
Reverse Kaplan–Meier method to demonstrate cohort follow-up, loss to follow-up and death. Deaths are indicated by hash marks. The vertical line represents the primary follow-up point of 1 year post stroke

Median observation time of the cohort was 399 days [interquartile range (IQR): 149–739]. Median loss to follow-up for survival and death (hash marks) in the cohort is shown by the reverse Kaplan–Meier method^22^ in Figure 1. Steep descents in the curve represent the follow-up windows at 90 days and at 1 year. The area under the curve is the total person-days of observation in the cohort, 213 862.

## What has been measured?

The register dataset was informed by the South London Stroke Register^23^ and standard international stroke register methods.^12^ During the initial admission the following data were collected; basic demographics, stroke risk factors, care seeking and pre-admission treatment, consultation fees and travel expenditure. Stroke severity was measured by the National Institute of Health Stroke Scale (NIHSS)^24^ and functional status was measured by Barthel Index^25^ and modified Rankin Scale (mRS).^26^ Health-related quality of life was measured by the Krio version of the EQ-5D-3L.^27^ Quality of care, hospital processes, in-hospital complications and final outcome were recorded.

Classification of pathological stroke subtype was performed by a stroke expert (A.R.); cases were classified using the Oxford Community Stroke Project (OCSP) classification^28^ and the Trial of Org 10172 in Acute Stroke Treatment (TOAST) classification.^29^ Classification was based on results from at least one of: brain imaging within 30 days of stroke onset [either by computed tomography (CT) or magnetic resonance image (MRI)]; or cerebrospinal fluid analysis in cases of suspected subarachnoid haemorrhage where brain imaging was not diagnostic. No patients underwent necropsy/autopsy. Cases without pathological confirmation of stroke subtype were classified as undetermined stroke type. The study had limited access to echocardiogram and carotid imaging and therefore we were unable to complete TOAST classification for many patients. Key variables, summary statistics, method of collection and missing item data counts are described in Table 2.

**Table 2.** Key variables, with method of collection and missing data

Variable	Summary statistic	Method of collection	Data missing or not collected (*n *=* *986)
Mean age (SD), years	58.9 (14.0)	Patient or caregiver reported and identification	0
Sex (male)	495 (50.2%)	Patient or caregiver reported	0
District (Western Area)	822 (84.1%)	Patient or caregiver reported	8 (0.8%)
Ethnicity	—	Patient or caregiver reported	6 (0.6%)
Higher educational attainment	367 (37.2%)	Patient or caregiver reported	24 (2.4%)
Employed (full- or part-time)	359 (38.2%)	Patient or caregiver reported	47 (4.8%)
Breadwinner	424 (43%)	Patient or caregiver reported	46 (4.7%)
Hypertension	831 (84.3%)	Patient reported and biochemical and physiological	0
Diabetes	212 (21.5%)	Patient reported and biochemical	0
Lipidaemia	401 (40.7%)	Patient reported and biochemical	1 (0.1%)
Smoking (current)	153 (15.8%)	Patient or caregiver reported	15 (1.5%)
Alcohol (any)	255 (26.7%)	Patient or caregiver reported	31 (3.1%)
Date of stroke onset	—	Patient or caregiver reported	1 (0.1%)
Sought care prior to hospital	456 (47.5%)	Patient or caregiver reported	26 (2.6%)
Median National Institute of Health Stroke Scale score (IQR)	16 (9–24)	Physician examination	3 (0.3%)
Median Barthel Index score before stroke (IQR)	100 (100–100)	Recall, scored by trained research assistant	6 (0.6%)
Median Barthel index score 7 days after stroke (IQR)	30 (0–50)	Observation, by trained research assistant	204 (20.7%)^a^
Median modified Rankin scale score 7 days after stroke (IQR)	5 (5–6)	Observation, scored by trained research assistant	135 (13.7%)^a^
Date of arrival	—	From medical chart	10 (1.0%)
Referred for physiotherapy	582 (59.8%)	From medical chart	13 (1.3%)
Statin prescribed	591 (60.4%)	From medical chart	8 (0.8%)
Antiplatelet prescribed for ischaemic stroke	403 (64.5%)	From medical chart	31 (3.1%)
Stroke type		Clinician, using charts and neuroimaging	0
Ischaemic	625 (63.4%)		
Intracerebral haemorrhage	206 (20.9%)		
Subarachnoid haemorrhage	25 (2.5%)		
Undetermined	130 (13.2%)		
In-hospital death	355 (36.0%)	From medical chart	0
In-hospital complications	396 (40.2%)	From medical chart	0
30-day case fatality	366 (37.1%)	From medical chart or caregiver reported	
90-day case fatality	438 (44.4%)	Caregiver reported	
1-year case fatality	492 (49.9%)	Caregiver reported	

Data are count (%), mean (SD) or median (IQR).

SD, standard deviation; IQR, interquartile range.

a Due to death occurring before 7 days or early discharge.

## Definition of risk factors and outcome measures

Participant ethnicity and ethnicity of both parents were recorded and categorized by the main tribal groups in Sierra Leone.^30^ Employment status, job and the highest educational attainment of the participant was recorded. Participants were asked if they were the main breadwinner for the family, defined as the principal income generator for the family. Time and date of stroke onset were defined as the onset of the first stroke symptoms; for cases of ‘wake-up stroke’, this was defined as the last time point the patient was witnessed to be symptom free. Participants were asked details of their care seeking before arriving at either of the two hospitals, their mode of transportation and the expenditure on transport, medicine, consultation and medication before arriving at the hospital.

Due to limited access to primary health care and low levels of health literacy and care seeking, for many participants their stroke admission is their first encounter with the formal health care system and they present with underlying undiagnosed risk factors. Therefore, we used risk factor definitions, see Table 3, which did not rely on accessing prior patient health records, in line with other stroke studies in the region.^7^^,^^31^

**Table 3.** Risk factor definitions used in the Stroke in Sierra Leone register

Condition	Classification
Hypertension	Blood pressure ≥140/90 mmHg from 72 h after stroke; or patient-reported history of hypertension; or history of antihypertensive use or continuing use of antihypertensives 72 h after stroke
Type 2 diabetes mellitus	Previous documented history of diabetes; or previous reported history of diabetes; or previous prescription of diabetic medication; or HbA1_c_ >6.5% on admission
Dyslipidaemia	Previous history of dyslipidaemia; or previous prescription of lipid-controlling drugs; or a fasting total cholesterol concentration of at least 5.2 mmol/L, HDL cholesterol of 1.03 mmol/L or lower, triglyceride of at least 1.7 mmol/L, or LDL cholesterol of at least 3.4 mmol/L on admission

HbA1_c_, glycated haemoglobin; HDL, high-density lipoprotein; LDL, low-density lipoprotein.

Participants were categorized as: smokers, ex-smokers or never smoked, smoking exposure being measured in pack-years; and as current/ex-/or never drinkers, with alcohol exposure calculated as units/week.

All participants who were stable for transfer, in total 857 (87%), received neuroimaging, either CT or MRI. All participants had venesection performed for full blood count, renal function, glycated haemoglobin (HbA1c) and lipid profile. HIV testing was conducted using the routine hospital HIV service. A single 12-lead electrocardiogram was performed for all patients. Cardiac Holter, echocardiography and carotid Doppler are not available routinely in government hospitals and were not conducted as part of the study.

Clinicians received formal training on National Institute of Health Stroke Severity Scale and performed scoring under supervision of co-investigators until proficient. All clinical research staff were trained on the use of the Barthel Index and modified Rankin Scale (mRS). Functional outcome was measured using the Barthel Index and mRS and was retrospectively reported by patients and family 7 days prior to stroke, then measured at 7 days post stroke, 90 days post stroke, 1 year post stroke and each year thereafter. Quality of life was measured using the interviewer-administered EQ-5D-3L in Krio for Sierra Leone at the same time points.

All-cause mortality was recorded from hospital records and as reported by caregiver or relative at follow-up. Readmission to hospital was recalled by patient or caregiver and monitored by active surveillance at the two hospitals.

## What has it found?

**Figure 2.**
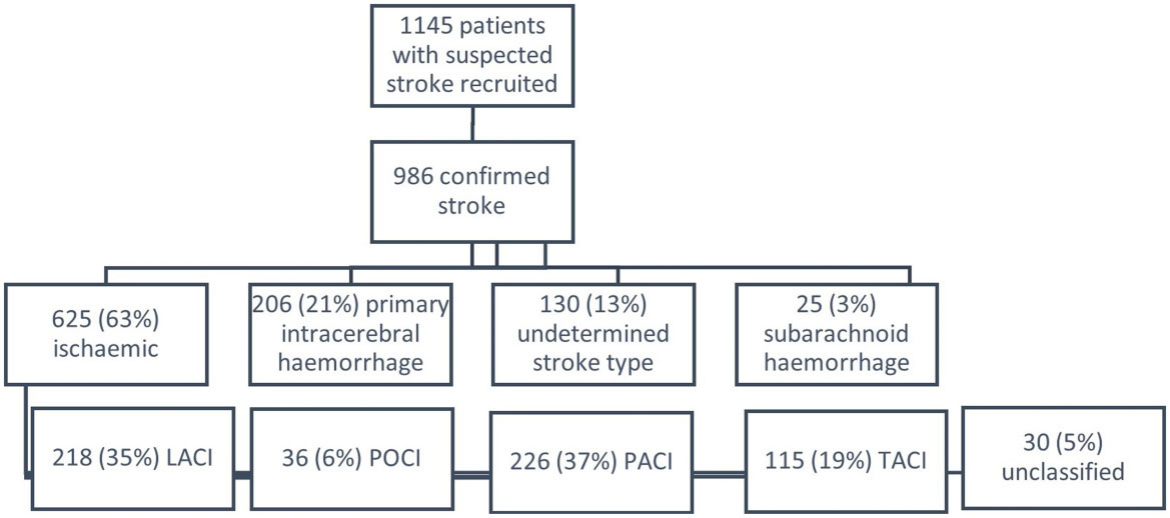
Flowchart of recruitment and stroke types by Oxford Community Stroke Project (OCSP) classification. TACI, total anterior circulation infarction; PACI, partial anterior circulation infarction; POCI, posterior circulation infarction; LACI, lacunar infarction

The SISLE stroke register has documented the natural history of stroke in Sierra Leone, including stroke case mix and stroke type, Figure 2. The register reports severe strokes, occurring in younger patients than those recoded in other settings,^32^ the majority of whom were previously fully independent regarding activities of daily living.^33^ Stroke cases were equally split by sex, mean age was 58.9 [standard deviation (SD): 14.0] years and the median NIHSS was 16 (9–24). The median time from stroke onset to admission was 24 h and median length of stay was 7 days (IQR 3–12).

**Figure 3.**
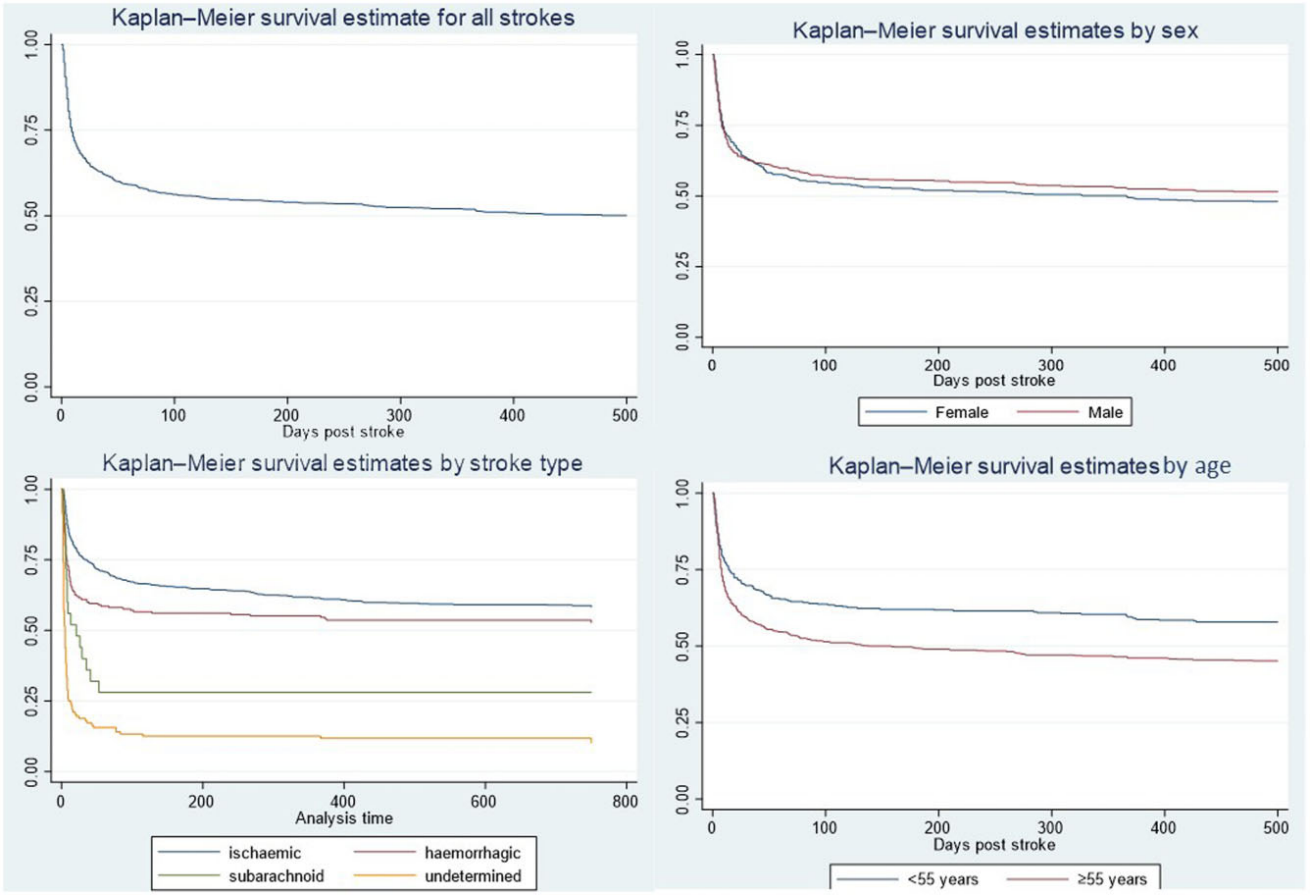
Kaplan–Meier estimates for stroke survival from date of stroke onset. Kaplan–Meier survival estimate for all patients (*n* = 986). Survival estimate by sex (*n* = 986), logrank test *P* = 0.75. Survival estimate by stroke type (*n* = 986), logrank test *P* = 0.0001. Survival estimate by age <55 years vs ≥55 years (*n* = 981), logrank test *P* = 0.0001

The SISLE register has documented the long-term outcomes for people with stroke for the first time in Sierra Leone. Case fatality rate was 37.1% at 30 days, 44.4% at 90 days, 49.9% at 1 year and 53.2% at 2 years.^34^ Kaplan–Meier survival estimates for all patients by sex, stroke type and age are shown in Figure 3.

SISLE register has demonstrated functional outcome and timing of recovery; 93% of participants were completely independent (Barthel Index score 100) prior to their stroke, declining to 19% at 1 year after stroke. Functional improvement was most likely to occur between 7 and 90 days post stroke, with 35% of all participants improving and 13% improving between 90 days and 1 year.^34^

The SISLE register examined the associations with increased fatality, which includes male sex, previous stroke, atrial fibrillation, subarachnoid haemorrhage, undetermined stroke type and in-hospital complications. The SISLE register has documented prevalence of risk factors in stroke patients: 841 (83%) had hypertension, 212 (21.5%) had type 2 diabetes mellitus, 579 (58.8%) had dyslipidaemia and 38 (3.9%) had atrial fibrillation.

The SISLE register assessed quality of care for stroke in Sierra Leone. Almost half, 49.6%, of all patients had a stroke-related complication. Pneumonia was the most prevalent complication, reported in 25.5% of patients.^33^ The high prevalence of aspiration pneumonia in the SISLE register dataset informed the development of a pilot randomized controlled trial of swallow screening, to prevent aspiration pneumonia.

The stroke register continued recruitment throughout the COVID-19 pandemic. During the first 3 months of the COVID-19 pandemic in Sierra Leone, April, May and June 2020, we saw a slight decrease in patient presentation at the hospital. We recruited 79 patients with confirmed strokes, compared with 98 patients with confirmed stroke in the following 3 months and 110 patients with confirmed strokes in the preceding 3 months. Median NIHSS was 15 (IQR: 9–23) during the first 3 months of COVID-19, compared with 14 (IQR: 10–23) in the following 3 months and 14 (IQR: 7–24) in the prior 3 months.

Further planned analysis will use the EQ-5D-3L to describe quality of life after stroke and variables associated with quality of life in this setting. Quality-adjusted life-years (QALYs) will be calculated using EQ-5D-3L and survival data from the register. Qualitative work from the register explores the experiences of stroke survivors, informal caregivers and health care providers in Sierra Leone.^35^ Further planned analyses include stroke in people living with HIV vs HIV-negative people, and sex differences in stroke and stroke outcome in Sierra Leone.

## What are the main strengths and weaknesses?

The SISLE register is one of the larger longitudinal prospective stroke registers in SSA.^4^^,^^36^^,^^37^ The cohort benefits from prospective methodology and clearly defined risk factors and outcome measures, generating internationally comparable data of stroke risk factors, stroke types and stroke outcomes. The study has a relatively high rate of neuroimaging (87%), and follow-up rates are 88.3% at 90 days and 81.5% at 1 year. The funding of stroke-related investigations likely reduced selection bias onto the register, perhaps evidenced by the gender parity in this register compared with other hospital-based cohorts in SSA.^4^ However, the register is not population-based, therefore results cannot be extrapolated to the population level and are influenced by access to care. Selection bias onto the register, with care seeking only for patients with severe strokes and under-detection of patients with less severe strokes, likely contributes to the high case fatality rate reported.

## Can I get hold of the data? Where can I find out more?

Data from the SISLE register are available to other researchers, and the SISLE researchers are interested in collaborating in more detailed and comparative research. Requests for data access for academic use should be made to the Kings College London (KCL) Stroke Research Group where data will be made available subject to academic review and acceptance of a data-sharing agreement. Requests should be made by e-mail to [registry.comahs@usl.edu.sl] and [stroke-register@kcl.ac.uk]. Request should include a 400-word scientific abstract with the following titles: Introduction, Scientific rationale, Methods, Results and Potential impact of the research. Requests should be accompanied by CV of the principal researchers. Requests will be reviewed and decisions communicated by the KCL Stroke Research Group within 6 weeks of submission date.

## Ethics approval

The study received ethical approval from Kings College London (HR-18/19–8467) and approval from the Sierra Leone Ethical and Scientific Review Committee on 18 December 2018. Written consent was sought from all patients. For those judged not to have capacity, informed consent was sought from the next of kin.

## Supplementary Material

dyad112_Supplementary_Data

## Data Availability

The data underlying this article will be shared on reasonable request, see ‘Can I get hold of the data?’ above.
