# Supplementary material for: Cohort Profile: The Stroke in Sierra Leone (SISLE) Register
Source: Int J Epidemiol. 2023 Aug 9;52(6):e308–14. doi: 10.1093/ije/dyad112 (PMC10749756; doi:10.1093/ije/dyad112)
Supplement: dyad112_Supplementary_Data [file dyad112_supplementary_data.docx]

**Cohort Profile: Stroke in Sierra Leone register - Supplementary Material**

**Table S1: Univariable count (%) by dead at one year, alive at one year and lost to follow up at one year**

|  | **Dead at one year N=492** | **Alive at one year N=312** | **Lost to follow up  at one year N=182** |
| --- | --- | --- | --- |
| Age mean (SD) | 61.2 (13.9) | 55.7 (13.9) | 58.4 (14.7) |
| Male | 238 (48.4%) | 151 (48.4%) | 106 (58.2%) |
| Resident of Western Area urban or rural | 422 (85.8%) | 263 (84.3%) | 137 (75.3%) |
| Hypertension | 401 (81.5%) | 271 (86.9%) | 159 (87.4%) |
| Diabetes | 122 (24.8%) | 61 (19.6%) | 29 (15.9%) |
| Higher education level (finished high school) | 170 (34.6%) | 128 (41.0%) | 69 (37.9%) |
| Pre-stroke Barthel Index Median score and IQR | 100 (100-100) | 100 (100-100) | 100 (100-100) |
| Mean National Institute of Health Stroke Severity Scale (SD) | 21.3 (8.6) | 11.5 (6.9) | 12.8 (7.6) |
| Stroke type: Ischaemic | 258 (52.4%) | 231 (74.0%) | 136 (74.7%) |
| Intracerebral haemorrhage | 95 (19.3%) | 70 (22.4%) | 41 (22.5%) |
| Subarachnoid | 18 (3.7%) | 6 (1.9%) | 1 (0.5%) |
| Undetermined | 121 (24.6%) | 5 (1.6%) | 4 (2.2%) |
| Median Barthel Index score at seven days post stroke (IQR) | 0 (0-25) | 40 (20-55) | 40 (25-60) |

SD=standard deviation IQR=Interquartile range
